# The effect of exposure to traffic related air pollutants in pregnancy on birth anthropometry: a cohort study in a heavily polluted low-middle income country

**DOI:** 10.1186/s12940-023-00973-0

**Published:** 2023-02-27

**Authors:** Frida Soesanti, Cuno S. P. M. Uiterwaal, Kees Meliefste, Jie Chen, Bert Brunekreef, Nikmah S. Idris, Diederick E. Grobbee, Kerstin Klipstein-Grobusch, Gerard Hoek

**Affiliations:** 1 Department of Child Health, Faculty of Medicine, Universitas Indonesia/Cipto Mangunkusumo General Hospital, Jakarta, Indonesia; 2 Julius Global Health, Julius Center for Health Sciences and Primary Care, University Medical Center Utrecht, Utrecht University, Utrecht, The Netherlands; 3 Environmental and Occupational Health Group Institute for Risk Assessment Sciences (IRAS), Utrecht University, Utrecht, the Netherlands; 4 Division of Epidemiology and Biostatistics, School of Public Health, Faculty of Health Sciences, University of the Witwatersrand, Johannesburg, South Africa

## Abstract

**Background:**

Ambient air pollution has been recognized as one of the most important environmental health threats. Exposure in early life may affect pregnancy outcomes and the health of the offspring. The main objective of our study was to assess the association between prenatal exposure to traffic related air pollutants during pregnancy on birth weight and length. Second, to evaluate the association between prenatal exposure to traffic related air pollutants and the risk of low birth weight (LBW).

**Methods:**

Three hundred forty mother-infant pairs were included in this prospective cohort study performed in Jakarta, March 2016–September 2020. Exposure to outdoor PM_2.5_, soot, NO_x_, and NO_2_ was assessed by land use regression (LUR) models at individual level. Multiple linear regression models were built to evaluate the association between air pollutants with birth weight (BW) and birth length (BL). Logistic regression was used to assess the risk of low birth weight (LBW) associated with all air pollutants.

**Results:**

The average PM_2.5_ concentration was almost eight times higher than the current WHO guideline and the NO_2_ level was three times higher. Soot and NO_x_ were significantly associated with reduced birth length. Birth length was reduced by − 3.83 mm (95% CI -6.91; − 0.75) for every IQR (0.74 × 10^− 5^ per m) increase of soot, and reduced by − 2.82 mm (95% CI -5.33;-0.30) for every IQR (4.68 μg/m^3^) increase of NO_x_. Outdoor air pollutants were not significantly associated with reduced birth weight nor the risk of LBW.

**Conclusion:**

Exposure to soot and NO_x_ during pregnancy was associated with reduced birth length. Associations between exposure to all air pollutants with birth weight and the risk of LBW were less convincing.

**Supplementary Information:**

The online version contains supplementary material available at 10.1186/s12940-023-00973-0.

## Introduction

Ambient air pollution has been recognized as one of the most important environmental health threats in the world today [1, 2]. An emerging body of evidence shows that air pollutant exposure in early life may affect pregnancy outcomes and the health of the offspring [3, 4]. Air pollutant exposure has been linked to fetal growth restriction [5], low birth weight [6], and prematurity [7]. A recent meta-analysis showed that exposure to carbon monoxide (CO), nitrogen dioxide (NO_2_) and particulate matter less than 10 and 2.5 μm (PM_10_ and PM_2.5_) was associated with reduced birth weight of 11.4–28.1 g and increased odds of low birth weight of 1.05 to 1.10 for relevant contrasts of these pollutants [8]. However, most of these studies were performed in high income countries (HIC).

In low- and middle-income countries (LMIC), attention has focused mostly on indoor air pollution from unvented cooking and heating stoves and to a more limited extent, environmental tobacco smoke [9]. Most of the evidence of health effects from air pollution has been obtained from studies on PM_2.5_ concentrations that are low to moderate on a global scale [1]. In 2019, annual population-weighted PM_2.5_ concentration were highest in the WHO South-East Asia Region, followed by the WHO Eastern Mediterranean Region [1]. The pattern for NO_2_ concentration is quite different from those of PM_2.5_ with the highest in Eastern Asia, the Middle East, North America and much of Europe [1]. Indonesia was reported to have a median PM_2.5_ concentration of 18.47 (95% CI: 15.58–22.90) μg/m^3^ for urban and rural areas combined and 19.11 μg/m^3^ for urban areas only in 2018 [10]. This report was based on a global satellite-based model and not developed specifically for traffic as the source of air pollution. Local detailed actual measurements for traffic-related air pollution have not been performed in Indonesia. Jakarta, the capital metropolis of Indonesia, may serve as a model of intense air pollution exposure, specifically traffic-related air pollution. Some 70–80% of total air pollution is considered due to traffic, with an estimated 16.1 million motorcycles and 4.3 million cars, and heavily polluting public transport vehicles in the greater Jakarta area [11].

The neonatal mortality rate in Indonesia has not substantially improved in the past two decades [12]. Other than the traditional risk factors, one of the possible reasons for poor neonatal outcomes may be environmental pollution, specifically air pollution. This issue is particularly relevant for Indonesia, which is currently undergoing a transition towards an industrialized country. Fast-growing industries, a huge increase in motor vehicle use, and lifestyle changes towards increased tobacco smoking may result in a cumulative escalation of environmental pollution in the face of inadequate pollution control. These local circumstances might affect the association between air pollution exposure and birth outcomes. Our study aimed to investigate the association between early exposure to traffic related air pollutants during pregnancy with birth anthropometry. Second, we explored the association of early exposure to outdoor air pollutants with the risk of low birth weight.

## Methods

This prospective birth cohort study was performed in Jakarta from March 2016 until September 2020. The study recruited 413 mothers in early pregnancy from nine primary health care centers (Cempaka Putih, Johar Baru, Kemayoran, Kramat, Jatinegara, Kampung Melayu, Matraman, Paseban, Rawa Bunga) in Jakarta, Indonesia. There were 69, 132, 122, and 90 pregnant women recruited in year 2016, 2017, 2018, and 2019, respectively. This study was ethically approved by the Institutional Review Board of the Faculty of Medicine University of Indonesia/Cipto Mangunkusumo General Hospital, Jakarta, Indonesia (reference number: 895/UN2.F1/ETIK/2015). Written informed consent was obtained from all the participants prior to study enrolment.

### Study population

Pregnant women were recruited in their first trimester or early in the second trimester of pregnancy (gestational age < 20 weeks) during their antenatal care visit. They were included in the study if they were residing in the catchment area of the primary care centers and could be contacted by phone. Recruitment was performed by midwives in each health care center. After enrolment, pregnant mothers were followed-up in line with routine antenatal care (ANC) guidelines and underwent several examinations, including anthropometrics, blood pressure, and ultrasound. We excluded (1) subjects who refused to continue their participation in this study and (2) subjects who moved out of town during follow up period. Participating women were followed up until delivery and their infants were followed up until the age of 6 months.

### Data collection

#### Assessment of demographic, socio-economic and pregnancy-related information

Maternal characteristics including maternal age, parity, history of abortion, working status, family income, level of education, any diseases prior to pregnancy, alcohol or illicit drug use prior to pregnancy, smoking history prior to pregnancy were obtained using structured questionnaires at enrolment. Paternal characteristics, including age, working status, level of education, and smoking habits prior to their spouse’s pregnancy were also obtained at recruitment.

A structured questionnaire was performed in every trimester to obtain any pregnancy complications and medications, current smoking habit, and exposure to indoor pollutant such as cooking fuel, insecticides, garbage burning, as well as dietary intake. Pregnant women who did not smoke but were exposed to household members who smoked on a daily basis at home in the presence of the pregnant women were considered as exposed to passive smoking, while pregnant women who were smoking throughout pregnancy were considered as active smoker. Data regarding the average number of cigarettes smoked per day throughout the duration of pregnancy for each smoker were recorded. Most of the pregnant woman had their delivery in primary care. Referral to the secondary health care center for complicated pregnancy or delivery was performed in accordance with the national health care policy.

Body weight, fundal height and blood pressure of the pregnant mothers were measured at every ANC visit. Weight gain during pregnancy (expressed as Δ BMI) was calculated using the formula of BMI at delivery-BMI prior to pregnancy. At the time of delivery, maternal blood pressure and body temperature were measured; gestational age and complications during pregnancy and delivery (preeclampsia/eclampsia, maternal infections, premature rupture of the membrane, gestational diabetes, or other delivery complications) were also recorded.

#### Infant outcomes measurements

Infant weight, length, and Apgar score were measured at birth. Birth weight and length were measured using a standardized protocol twice and then averaged. The averaged value was used for the final analysis. Infants who developed complications were examined by the attending physician and received standard care including referral to secondary health care center. Neonatal morbidities, including respiratory distress, asphyxia, bacteremia/sepsis, hyperbilirubinemia, and congenital anomalies and all causes of neonatal mortality were recorded. Neonatal mortality was defined as any mortality occurring within the first 28 days after birth. Other adverse birth outcomes were defined as preterm birth if gestational age < 37 weeks and low birth weight if birth weight < 2500 g [13].

#### Outdoor air pollution assessment

**Fig. 1:**
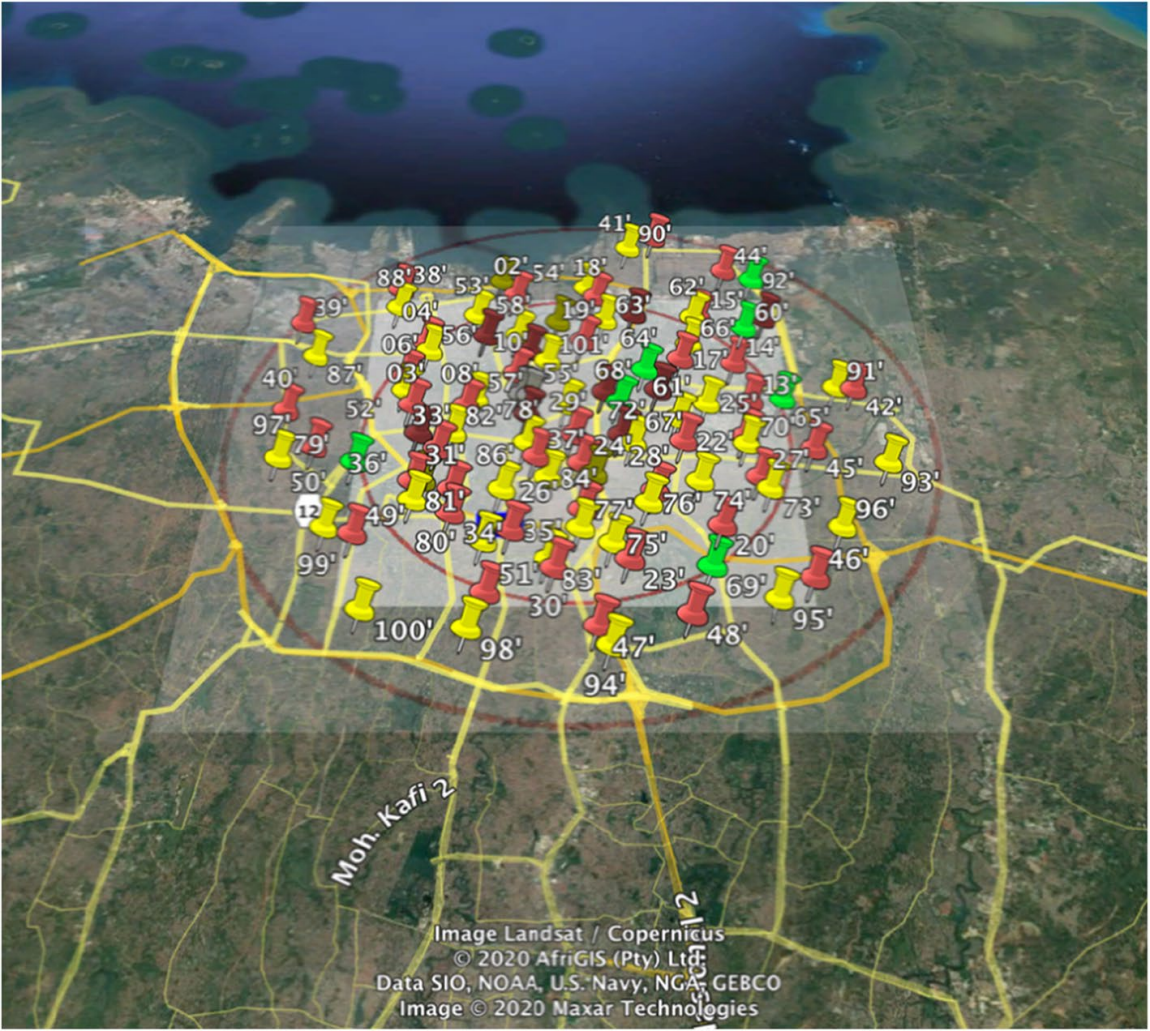
Study area of Jakarta air pollution sampling. Red pin indicates traffic sites, yellow pin: urban background, green pin: urban green, white pin indicates reference site

Exposure assessment for the cohort was based upon land use regression (LUR) models [14] developed based on targeted measurements of fine particles and nitrogen oxides in Jakarta. The study area was defined by the primary care catchment area of the cohort study. The study area is part of the center of Jakarta (Fig. 1). LUR models were developed for particles smaller than 2.5 μm (PM_2.5_), soot (a measure of black carbon), nitrogen dioxide (NO_2_) and the sum of nitrogen dioxide (NO_2_) and nitrogen oxide (NO), denoted as NO_x_. Measurements were made at 88 sites across the study area. We selected 37 urban background sites, 7 urban green sites and 44 traffic sites, based upon the assumption that motorized traffic is an important source of spatial variability in the study area. Annual average concentrations were calculated after correction for temporal variation with continuous measurements from a reference site [14].

LUR models were developed using predictor variables obtained from direct systematic field observations of traffic counts and street configuration and global GIS databases of road data from Open Street map and impervious surface (Refer to Additional file 1). Models were developed using supervised linear regression procedures, extensively used in previous LUR studies [15]. The developed LUR models are listed in Supplemental Table 1. All models included motorcycle counts at the nearest road as an important predictor variable. The LUR models explained 61, 59, 26 and 33% of the measured annual average concentration variability for NO_x_, NO_2,_ PM_2.5_ and soot respectively. The models thus explain a moderate amount of the measured variability. Exposure to air pollution was assessed at individual level, calculated for each member of the cohort by applying the LUR model to the residential address [16].

Because of the lack of continuous monitoring data for all four evaluated pollutants, we could not use extrapolation methods. Continuous monitoring data was available from the US embassy only for PM_2.5_ [17]. We downloaded hourly data from 2016 to 2020 for the central Jakarta site and calculated annual average to evaluate a possible trend over time. We further evaluated variability of monthly averages and calculated full pregnancy average concentrations related to temporal variation. We also measured indoor air pollutants concentration for PM_2.5_, soot, NO_x_, NO_2_ in a subset of 47 randomly selected participants (Refer to Additional file 1).

### Statistical analysis

Baseline characteristics were tabulated to describe general characteristics of the mothers and infants, as well as for smoking habits and other environmental factors such as home fuel resources usage, garbage burning, and household insecticides usage. Continuous variables were expressed as mean and standard deviation or median and interquartile range if distributions were skewed. Categorical variables were expressed as number of subject and its percentage.

Socio-economic status (SES) i.e. household income and level of education, maternal age, parity [6, 18, 19], BMI increment during pregnancy (Δ BMI) [19], mother’s working status, mother’s active and passive smoking during pregnancy [8, 19], and pesticides exposure in pregnancy were a priori considered as possible confounders. All of the possible confounders were treated as categorical, excluding mother’s age and Δ BMI. Level of education was categorized as elementary, high school, and under-post graduate, while family income was categorized as below the minimum monthly wedges per capita in Jakarta (< 290 USD) or above or equal to the minimum monthly wedges per capita in Jakarta (≥ 290 USD). Maternal co-morbidity/gestational complication [20] and infant gender were considered as possible effect modifiers [18, 19].

Multiple linear regression models adjusted for gestational age and subsequently all potential covariates were specified to evaluate the association between outdoor concentration of PM_2.5_, soot, NO_x_, and NO_2_ with birth weight and birth length. Logistic regression was used to explore the association between outdoor air pollutants concentration with low birth weight, expressed as OR with 95% confidence interval. We performed sensitivity analysis to test the robustness of our results when restricting the cohort to women with infant born at gestational age ≥ 37 weeks. We additionally calculated the difference between PM_2.5_ and soot to further differentiate effects of PM_2.5_ and soot. This difference was highly correlated (R = 0.99) with PM_2.5_. We also isolated the effect of soot from PM_2.5_ by generating new variable as the residual of soot [21] and further, we tested the association of this new variable (the residual of soot) with birth anthropometry. All effect estimates were expressed for an interquartile increase of each air pollutant i.e. 7.14 μg/m^3^ for PM_2.5_, 0.75 × 10^− 5^ per m for soot, 4.68 μg/m^3^ for NO_x_, and 3.74 μg/m^3^ for NO_2._ Statistical significance was assumed if 95% confidence intervals did not include the estimation of null values, corresponding to two-sided *p* values < 0.05. Statistical analyses were conducted using IBM SPSS version 24 for Mac, while the LUR models were developed using R 4.1.2.

## Results

Initially, 413 pregnant women were enrolled in this study, yet 73 mother-infant pairs were not included in the analysis i.e. 52 dropped for various reasons (refused to continue or moved out of town), 13 experienced miscarriages at an early stage of the pregnancy, one subject had ectopic pregnancy, and 7 had incomplete birth anthropometric measurement. Further, one baby with extremely low birth weight (GA 27 weeks, BW 700 g) was excluded. In the end, 340 mother-infant pairs were included in this study.

The pregnant women in our study were 27.9 (SD 5.37) years of age on average, most were high school graduates and on their first pregnancy (Table 1). Around 41.2% of pregnant women worked during pregnancy and 27.1% had pregnancy complication. Infants were born with mean gestational age of 38.6 (SD 1.58) weeks, birth weight of 3117.7 (SD 427.2) g, and birth length of 48.3 (SD 1.98) cm. The first infant was born on 13th October 2016. Twenty one infants had low birth weight and 18 of them were prematurely born. Most fathers smoked (63.8%) during pregnancy, while only 7 women actively smoked.

**Table 1:** Baseline subject characteristics

Variable	Total (*n* = 340)
**FAMILY CHARACTERISTICS**	
Mother age (years)	27.92 (5.37)
Mother’s enrolment period, n (%)	
• Trimester 1	165 (48.5)
• Trimester 2	175 (51.5)
Parity, n (%)	
• 0–1	233 (68.5)
• **≥** 2	107 (31.5)
Without history of abortion, n (%)	274 (80.8)
Mother’s education level, n (%)	
• Elementary	21 (6.1)
• High school	244 (71.8)
• Under-post graduate	75 (22.1)
Family income (USD/month), n (%) > 290 USD	121 (36.1)
Working during pregnancy, n (%)	140 (41.2)
BMI increment by pregnancy (kg/m^2^)	4.99 (3.04)
Pregnancy complications:	
• Hypertensive-preeclampsia, n (%)	23 (6.8)
• PROM, n (%)	46 (13.5)
• Oligohydramnion, n (%)	23 (6.7)
**INFANTS**	
Male sex, n (%)	169 (49.7)
Gestational age (weeks)	
• FDLM	38.58 (1.58)
Mode of delivery, n (%)	
• C-section	154 (44.8)
Birth weight (grams)	3117.7 (427.2)
Birth length (cm)	48.30 (1.98)
Low birth weight (BW < 2500 g), n (%),	21 (6.3))
• Weight (g), range	(1500–2475)
Premature (GA < 37 weeks), n (%)	18 (5.23)
• GA (weeks), range	34.33 (30–36)
**SMOKING HABITS**	
Mother who actively smoking during pregnancy, n (%)	7 (2.1)
Father smoking status during pregnancy, n (%)	215 (63.8)
Number of other household smoking during pregnancy, n (%)	
• 1 member	155 (45.7)
• **≥** 2 members	88 (26.1)
Total cigarettes/day	13.46 (9.99)
**ENVIRONMENT**	
Home fuel resources, n (%)	
Gas	277 (81.5)
Kerosene or others	7 (2.1)
Household insecticides usage, n (%)	151 (49.2)
Routine garbage burning, n (%)	8 (2.3)
Motorcycle density on nearest street (vehicle/day)	243.8 (0–47,731.7)

Numbers are expressed in mean (SD) otherwise indicated

*PROM* premature rupture of the membrane, *FDLM* first day of last menstrual period, *GA* gestational age, total cigarettes/day: average total cigarettes consumed by the household member per day

Table 2 shows the air pollutant concentration both from outdoor and indoor assessment. The mean of outdoor PM_2.5_ concentration was almost eight times (36.54 μg/m^3^) as high as the recommended WHO guideline level (5 μg/m^3^ annually) [1]. The mean concentration of outdoor NO_2_ were at least three times higher (32.62 μg/m^3^) than the recommended WHO guideline level of 10 μg/m^3^ annually [1]. The average concentration of all indoor air pollutants were higher than the respective outdoor concentrations. Table 2 illustrates similar moderate contrast of exposure within the cohort for the four evaluated pollutants. The standard deviation is about 10% of the mean for all pollutants (Table 2) and the correlation between the pollutants was weak except for the correlation between soot with PM_2.5_ which was moderate (0.51), and the correlation between NO_x_ with NO_2_ which was high (0.87) (Supplemental Table 2 and 3). Based on the continuous PM_2.5_ monitoring data from the US embassy, the annual average concentrations of PM_2.5_ in central Jakarta were 40, 28, 38, 40 and 34 μg/m^3^ in 2016, 2017, 2018, 2019 and 2020 respectively, showing no trend in this short period. Monthly averages ranged between 15 and 58 μg/m^3^. Full pregnancy period averages calculated for infants conceived between January 2016 and December 2020 ranged between 26 and 45 μg/m^3^, with the lower values for those conceived in 2017.

**Table 2:** Distribution of the air pollutants concentration levels (outdoor and indoor) in the full cohort (*n* = 340) and the indoor measurements cohort (*n* = 47)

Air pollutant	Outdoor air pollutants (***n*** = 340) Mean ± SD (min-max)	Outdoor-indoor air pollutants (***n*** = 47)	
		Outdoor Mean ± SD (min-max)	Indoor Mean ± SD (min-max)
PM_2.5_ (μg/m^3^)	36.54 ± 3.75 (32.45–51.28)	37.40 ± 3.90 (33.83–47.28)	57.79 ± 38.46 (17.03–250.54)
Soot (10^− 5^ per m)	4.57 ± 0.54 (3.74–6.37)	4.53 ± 0.51 (3.74–5.59)	6.72 ± 3.15 (2.17–18.45)
NOx (μg/m^3^)	37.15 ± 4.24 (23.25–54.61)	36.68 ± 4.34 (27.97–43.07)	69.92 ± 31.23 (27.75–209.87)
NO_2_ (μg/m^3^)	32.62 ± 3.29 (21.30–44.84)	32.35 ± 3.36 (25.58–37.45)	56.81 ± 21.06 (15.00–108.11)

Table 3 shows the association between exposure to outdoor air pollutants during pregnancy with birth anthropometrics. Outdoor soot and NO_x_ were significantly associated with reduced length at birth in fully adjusted regression model. The birth length was significantly reduced by − 3.83 mm (95% CI -6.91; − 0.75) for every 0.74 × 10^− 5^ per m (IQR) increase of soot, and significantly reduced by − 2.82 mm (95% CI -5.33;-0.30) for every 4.68 μg/m^3^ (IQR) increase of NO_x_ concentration. We also observed an inverse association between PM_2.5_ and NO_2_ with birth length, but those association were not significant statistically. The beta coefficient of the final model (model 2) of the association between the residual of soot [21] with birth length was − 2.91 mm (95%CI of − 6.37;0.55) per every IQR increase of soot. This results showed that the association between soot and reduced birth length is independent of PM_2.5_.

**Table 3:** Association between exposure to outdoor air pollutants during pregnancy with birth anthropometrics

	Linear regression coefficients (95% confidence interval)	
	Birth weight (g) (***n*** = 340)	Birth length (mm) (***n*** = 340)
PM_2.5_		
Crude	−52.34 (−150.24; 45.57)	− 3.43 (− 7.93; 1.08))
Model 1	− 36.84 (− 126.91; 53.23)	− 2.89 (− 7.22; 1.44)
Model 2	− 54.44 (− 146.14; 37.27)	−3.45 (− 7.87; 0.97)
Soot		
Crude	−47.02 (− 116.78; 22.74)	− 3.60 (− 6;80; − 0.40)*
Model 1	−46.75 (− 110.77; 17.69)	− 3.60 (− 6;67; − 0.54)*
Model 2	−48.76 (− 113.15; 15.63)	−3.83 (− 6;91; − 0.75)*
NO_x_		
Crude	−5.16 (− 61.05; 50.74)	−2.18 (− 4.74; 0.39)
Model 1	−6.86 (− 58.18; 44.45)	−2.24 (− 4.70; 0.21)
Model 2	−17.20 (− 69.79; 35.39)	− 2.82 (− 5.33; − 0.30)*
NO_2_		
Crude	−4.28 (− 61.44; 52.87)	−1.35 (− 4.33; 1.63)
Model 1	−6.45 (− 58.92; 46.03)	−1.48 (− 4.44; 1.47)
Model 2	−17.20 (− 70.60; 36.02)	−1.91 (− 4.98; 1.17)

All effect estimates correspond to interquartile increase of each air pollutant i.e. every 7.14 μg/m^3^ for PM_2.5_, 0.75 × 10^− 5^ per m for soot, 4.68 μg/m^3^ for NO_x_, and 3.74 μg/m^3^ for NO_2_

Model 1: Adjusted for gestational age

Model 2: Adjusted for gestational age, parity, SES, mother’s working status, mother’s age at pregnancy, delta BMI during pregnancy, infant’s sex, passive smoking exposure, pregnancy complication

^*^p < 0.05

All outdoor air pollutants (PM_2.5_, soot, NO_x_, NO_2_) were inversely but not statistically significantly associated with birth weight in fully adjusted regression models. Birth weight was reduced by 54.4 g (95% CI -146.14;37.27) for every 7.14 μg/m^3^ increase in PM_2.5_, 48.76 g (95% CI of − 113.15;15.63) for every 0.75 × 10^− 5^ per m soot, and 17.20 g (95% CI -184.2;158.8 g) for every 3.74 μg/m^3^ increase in NO_2_ concentration. There was a positive association between all outdoor air pollutants concentration (PM_2.5_, soot, NO_x_, NO_2_) with the risk of LBW as shown in Table 4, but none of these were statistically significant. We found inconsistent result on the association between the indoor air pollutants with birth weight and birth length (Supplemental Table 4).

**Table 4:** Association between exposure to outdoor air pollutants during pregnancy with low birth weight

Outdoor air pollutant (***n*** = 340)	OR (95% confidence interval)
**PM**_**2.5**_	
Crude	0.88 (0.35; 2.19)
Adjusted	1.04 (0.31; 3.46)
**Soot**	
Crude	1.34 (0.73; 2.48)
Adjusted	1.73 (0.78; 3.83)
**NO**_**x**_	
Crude	1.12 (0.67; 1.86)
Adjusted	1.33 (0.68; 2.59)
**NO**_**2**_	
Crude	1.09 (0.65; 1.84)
Adjusted	1.10 (0.58; 2.06)

All effect estimates correspond to interquartile increase of each air pollutant i.e. every 7.14 μg/m^3^ for PM_2.5_, 0.75 × 10^− 5^ per m for soot, 4.68 μg/m^3^ for NO_x_, and 3.74 μg/m^3^ for NO_2_

Adjusted for gestational age, parity, SES, mother’s working status, mother’s age at pregnancy, delta BMI during pregnancy, infant’s sex, passive smoking exposure, pregnancy complication

Sensitivity analysis was performed to the determine the consistency of our results. When restricting the cohort to comprise only infants with term gestational age (GA ≥ 37 weeks), the association between outdoor air pollutants with birth anthropometric was robust (Table 5). The birth length reduction associated with soot and NO_x_ was consistent. The weight reduction was more pronounced but remain not significant statistically.

**Table 5:** Sensitivity analysis of association between exposure to outdoor air pollutants during pregnancy with birth anthropometrics (restricted to those with GA ≥37 weeks)

	Linear regression coefficients (95% confidence interval)	
	Birth weight (g) (***n*** = 319)	Birth length (mm) (***n*** = 319)
**PM**_**2.5**_		
Crude	−48.19 (− 137.14; 40.4)	− 2.75 (−7.01; 1.51))
Model 1	−36.42 (− 123.52; 50.68)	− 2.46 (− 6.71; 1.80)
Model 2	−65.09 (− 153.42; 23.23)	− 2.86 (− 7.24; 1.52)
**Soot**		
Crude	−36.60 (−99.67; 26.46)	− 3.55 (− 6;55; − 0.55)*
Model 1	− 36.58 (− 98.11; 24.95)	−3.56 (− 6;54; − 0.57)*
Model 2	− 39.15 (− 100.70; 22.41)	−3.78 (− 6;79; − 0.76)*
**NO**_**x**_		
Crude	−22.29 (− 74.87; 30.29)	−2.03 (− 4.42; 0.37)
Model 1	− 24.97 (− 76.55; 29.30)	−2.23 (− 4.63; 0.17)
Model 2	−38.11 (− 90.46; 14.22)	− 2.93 (− 5.38; − 0.48)*
**NO**_**2**_		
Crude	− 21.49 (− 75.15; 31.17)	−1.97 (− 5.03; 1.09)
Model 1	− 24.03 (− 75.05; 30.99)	−2.01 (− 5.08; 1.07)
Model 2	−37.48 (− 90.44; 15.48)	− 2.50 (− 5.66; 0.65)

All effect estimates correspond to interquartile increase of each air pollutant i.e. every 7.14 μg/m^3^ for PM_2.5_, 0.75 × 10^− 5^ per m for soot, 4.68 μg/m^3^ for NO_x_, and 3.74 μg/m^3^ for NO_2_

Model 1: Adjusted for gestational age

Model 2: Adjusted for gestational age, parity, SES, mother’s working status, mother’s age at pregnancy, delta BMI during pregnancy, infant’s sex, passive smoking exposure, pregnancy complication

^*^p < 0.05

## Discussion

Our study provides evidence regarding the effect of exposure to traffic related air pollution during pregnancy on birth anthropometry in low-middle income countries. Exposure to soot and NO_x_ was significantly and consistently associated with reduced length at birth. All air pollutants (PM_2.5_, soot, NO_x_, NO_2_) were also associated with reduced birth weight though not statistically significant. Confidence intervals for associations with the risk of low birth weight were wide. Few studies have explored the effect of soot on birth anthropometric and to our knowledge, ours is the first study in low-middle income countries to include the role soot as one of the important determinants of air pollution.

Birth length has been associated with intrauterine well-being of the fetus. Newborns are considered as small for their gestational age (SGA) if their birth length is less than 10th percentile according to their gestational age. Babies born SGA have negative long term outcomes including the risk of early cardiovascular diseases, diabetes, obesity [22], early puberty, and short stature [23]. Ballester et al. [24], reported that exposure to NO_2_ > 40 μg/m^3^ during the first trimester was associated with a 2,7 mm (95% CI -5.1; − 0.3) reduction in birth length. A study in Brisbane Australia, reported that IQR increase in NO_2_ (11.1 μg/m^3^) during the third trimester was associated with a reduction in crown-heel length of − 1.5 mm (95%CI -2.5;-0.5) [25]. However, Van Den Houven et al. [26] in the Netherlands did not find the same results. Clemens et al. [27] found birth length reduction of − 0.8 mm (95%CI -1.3;-0.3) associated with NO_2_ and − 2.3 mm (− 4.0;- 0.5) associated with PM_2.5_, while Jedrychowski et al. [28] also found − 10.0 mm reduction in birth length in the newborns of mothers who were exposed to PM_2.5_ above 36.3 μg/m^3^. Our result is consistent with these studies, to an extent that the effect on the birth length reduction is more pronounced in our study. Every IQR increase in NO_x_ (4.68 μg/m^3^) concentration result in birth length reduction of − 2.82 mm(95% CI -5.33;-0.30). Even though we did not observe the same result for NO_2_, but our findings expand observations on NO_2_ to NO and other nitrous compound that build NO_x_ on the birth anthropometric. NO_x_ is more related to primary combustion emissions, where NO_2_ also has a substantial secondary component (formation in the atmosphere once emitted).

We also found a consistently inverse association of prenatal exposure to soot on birth length, every 0.74 × 10^− 5^ per m increase of soot result in − 3.83 mm reduction in birth length and this association is independent of PM_2.5_ concentration. To our knowledge none of the previous studies focus on the effect of soot on the birth outcomes and birth anthropometric. Soot is elemental carbon emitted by fuel combustion of gas and diesel engines/ vehicles such as motorcycles, cars, and trucks. It has been postulated that (nano)particles, including soot can pass the placental barrier. However, most of the studies originated from in vitro cell cultures or animal studies. The recent evidence on soot translocation to human placenta following inhalation under real-life comes from the ENVIRONAGE birth cohort study [29]. The ENVIRONAGE study examined the presence of soot in placental tissue from a subset of their subjects, consist of 20 healthy, non-smoking women exposed to low (0.63–0.96 μg/m^3^) and high (1.70–2.42 μg/m^3^) levels of residential soot during pregnancy [29]. Soot particles were detected in all placentae, on both the maternal and fetal sides of the placenta, suggesting that black carbon may be transported to the developing fetus. The results of ENVIRONAGE study represents a potential mechanisms explaining the detrimental health effects of pollution from early life, including birth outcomes [29].

Although not statistically significant, the reduction in birth weight in our study were greater than those previously reported by other studies in high income countries or other Asia-Pacific countries. Similar results were observed by Augilera et al. [30] in the INMA cohort study. Dugandzic et al. [31] reported that an interquartile increase of exposure to PM_2.5_ and NO_2_ during pregnancy resulted in 14.7 g and 8.9 g reduction in birth weight. Malmqvist et al. [19] reported 9 g reduction in birth weight every 10 μg/m^3^ increase of NO_x_, while Steib et al. [4] in Canada reported a 16.2 g reduction (95% CI 13.6;18.8) per 20 ppb (37.6 μg/m^3^) NO_2_ in birth weight of term infant.

We did not find significant association between all outdoor traffic related pollutants with the risk of LBW although the ORs were slightly higher than previously reported. Likely, the wide confidence interval is due to small number of infants with LBW (*n* = 21). Liu et al. [32] reported the ORs of LBW was 1.06 (95% CI 1.02;11.0) for PM2.5 and 1.18 (95% CI 1.06;1.32) for NO_2_. The ESCAPE study reported a OR of LBW for every 5 μg/m^3^ increase in PM_2.5_ of 1.18 (95% CI 1.06;1.33) and the OR was 1.09 (95% CI 1.00;1.19) for every 10 μg/m^3^ increase in NO_2_ [33]. Other studies in HIC also reported similar result [6, 8, 34, 35].

The concentration of all outdoor and indoor air pollutants in this study was significantly higher than those measured in the birth cohort studies in high income countries i.e. the ESCAPE study (PM_2.5_ 36.54 vs 16.5 μg/m^3^, Soot 4.57 vs 1.7 10^− 5^ per m, NO_2_ 32.62 vs 26.2 μg/m^3^) [33]. The mean concentration for PM_2.5_ in this study was 8 times as high as the recommended level by WHO and the mean NO_2_ level was 3 times higher than the WHO recommendation [1]. This result shows the poor air quality in Jakarta, a metropolitan city in a LMIC characterized by urbanization and industrialization. The PM_2.5_ concentration that we measured between 2016 and 2018 (36.5 μg/m^3^) was very similar to the average concentration measured at a central site by the US embassy (35.2 μg/m^3^). Correlations between the four pollutant exposures were lower than observed in previous European studies [36], possibly because of a wider diversity of combustion sources in Jakarta. This may also be the reason for the somewhat lower model performance statistics of the LUR models for Jakarta compared to the European ESCAPE project [36]. We note that the generally low correlation was also found for measured concentrations at the monitoring sites and is therefore not an artefact from the modelling approach.

The biological mechanisms underlying the association between air pollutant and birth anthropometrics are not entirely understood. The pregnant women and her unborn child are considered to be more vulnerable towards exposure to air pollution due to pregnancy-related physiological adaptions and the intense prenatal growth and development [37]. Air pollution may trigger systemic, pulmonary and placental inflammation, oxidative stress, endothelial and cardiovascular changes which may result in pregnancy-induced hypertensive disorders, decreased trans-placental nutrient and gas exchange and thereby restrict the intrauterine growth of the placenta and the fetus [7, 37, 38]. A study by Lee et al. [39] showed that a 4.6 μg/m^3^ increase in PM_2.5_ was associated with an odds ratio of 1.47 of increased c-reactive protein (an inflammatory marker) during early pregnancy. Veras et al. [40] observed that in mice, PM exposure during pregnancy induced changes in multiple placental compartments, including maternal vascular space, fetal capillaries, and surface exchange areas. Mounting evidence showed that maternal exposure to air pollution can influence the placenta, where they alter DNA methylation patterns, leading to changes in placental function and fetal reprogramming. The ENVIR*ON*AGE birth cohort study showed that soot particles were detected on the maternal and fetal sides of the placenta, suggesting that soot may be transported to the developing fetus [29]. Another study by Liu et al. [41] determined the presence of air pollution nanoparticles in placental tissue cells that were isolated from 15 healthy, non-smoking women exposed to PM_2.5_ and PM10 during pregnancy.

Studies in animal models showed that exposure to NO_2_ during pregnancy induces lipid peroxidation in the placenta and disturbs postnatal development. NO_2_ may also have direct toxic effects on the fetus [7]. A study in a highly polluted area with NO_2_ in Bulgaria [42] suggested that a higher percentage of women in this area suffered from pregnancy complications, including preeclampsia, threatened abortion/premature labor and anemia. In this study, methemoglobin was significantly elevated in all women with pregnancy complication compared to women with normal pregnancy. This suggest that exposure to air pollution can increase the risk of pregnancy complications through stimulation of methemoglobin formation, which may lead to hypoxia and hypoxemia in pregnant women which can affect placental and fetal development [42].

We consider our prospective cohort study design with detailed air pollution measurements to be a major strength of this study. To date most of the epidemiological studies on the effect of air pollution to birth anthropometrics come from secondary data and retrospective trace of the exposure in the same area. To best of our knowledge this was the first study assessing the effect of early life exposure to air pollutants on birth anthropometrics in Indonesia and other LMIC in South-East Asia using LUR modelling to assess the individual exposure to all important air pollutants (PM_2.5_, soot, NO_x_, NO_2_) [30, 43, 44]. LUR estimates in this study were based on the women’s residential addresses to reduce the possibility of exposure misclassification [24, 30]. The prospective study design enrolling women in the first trimester of their pregnancy and following them and their offspring until the age of 6 months ensured comprehensive prospective data collection including quantitative measures of maternal active and passive smoking as well as maternal weight and height [24, 30].

Our study limitations relate to the relatively small sample size, potentially lacking the robustness to explore possibly subtle effects of early life exposure of air pollutants on birth anthropometrics. Technical errors in anthropometric measurements were minimized by performing the measurements twice, but might not fully be excluded, if any technical errors occurred we assume this was independent of the exposure. We did not measure the postnatal air pollution exposure, but Esplugues et al. [45] indicates that the air pollution exposure during the first year of life was highly correlated with antenatal exposure. Thus, we might confidently assume that the postnatal exposure to air pollution in our study was not different from the antenatal exposure. As there was no routine monitoring station providing daily concentrations, we could not calculate pregnancy or trimester-specific exposures. Our exposure contrast is based upon spatial differences in annual average concentrations. Other limitations was that LUR models in our study were generated based on the residential addresses and did not take into account the unknown occupational addresses. We cannot fully exclude the possibility of a non-differential misclassification in exposure assessment. This non-differential misclassification in exposure assessment could result in a decreased statistical power of our study and probably it would drive our study towards the null hypothesis. Exposure assigned at the residential address only is a very common issue in cohort studies in general, as the work address is generally not known.

In summary, the result of our study provides convincing epidemiological evidence that antenatal exposure to traffic related air pollution in pregnant woman result in adverse birth outcomes. Our study showed that soot and NO_x_ consistently results in birth length reduction. Our study may have implications not only for the health and development of children but also for adult health. Epidemiologic studies showed that prenatal hazards that restrict fetal growth may be associated with delays in motor and social development through childhood [46], reduced cognitive development [46], early puberty, short stature [23] and also for future development of adult diseases, such as type 2 diabetes and coronary artery disease [22]. This finding warrants further in depth investigation on a larger scale and implementation of policy changes, such as limiting the number of motor vehicles in major street, strict policy in motor vehicles trading, and emission measurement regularly to reduce the harmful effect of heavy air pollution for the developing fetus.

## Supplementary Information

**Additional file 1.** Methods of outdoor air pollutant measurement.

**Additional file 2: Supplemental Table 1.** LUR models for air pollutant concentration assessment.

**Additional file 3: Supplemental Table 2.** Correlation between air pollutants concentration at the measurement sites.

**Additional file 4: Supplemental Table 3.** Correlation between air pollutants concentration in the cohort.

**Additional file 5: Supplemental Table 4.** The association between exposure to indoor air pollutants during pregnancy with birth anthropometrics in a-indoor subset group.

## Data Availability

The datasets generated and/or analysed during the current study are not publicly available because the datasets contain multiple sensitive identifiers, but the datasets are available from the corresponding author on reasonable request.
