# Supplementary material for: The effect of exposure to traffic related air pollutants in pregnancy on birth anthropometry: a cohort study in a heavily polluted low-middle income country
Source: Environ Health. 2023 Feb 27;22:22. doi: 10.1186/s12940-023-00973-0 (PMC9969650; doi:10.1186/s12940-023-00973-0)
Supplement: Supplementary file 1 — Additional file 1. Methods of outdoor air pollutant measurement. [file 12940_2023_973_MOESM1_ESM.docx]

**Supplemental file 1. Methods of outdoor air pollutants assessment**

## Study area description

The study area covers the central part of Jakarta (**Figure 1).** The area is mostly non-industrial. Natural gas is used for cooking primarily. Heating does not occur because of the climate. Richer people use some air conditioners, most people us fan, also some electric cooking is used. The small streets (gangs) are too narrow for cars. The “steeg” is accessible for cars but often only used for motorbikes. Traffic consists of 50% motorbikes and 50 % of cars. Most cars are reasonably new and less polluting. Altitude differences are minimal. Jakarta has no typical season, there is no real dry and/or rainy season. The dry season usually start form March or May to September, while the wet/rainy season will start in October and end in March.

## Air pollution measurement data

*2.1. Site selection and study period*

PM_2.5_ and NO_2_/NO_x_ was measured between May 25, 2016 and May 2, 2018. In total, 88 sampling sites were selected. We selected urban background and traffic sites, to capture the relevant range in pollution concentrations. Background sites were defined as carrying less than 3000 vehicles per day with no other local sources, such as gas stations, small industries or large parking lots within 100 m. Traffic sites were defined as streets carrying more than 10,000 motor vehicles per day. The sites were furthermore spread over the study area (Fig. 1). Each selected site was measured two times for 14 days, spread over the study period. For each site, results from the two measurements were averaged to estimate the annual average, adjusting for temporal variation using a centrally located background reference site, which was operated for the entire sampling period. At the reference site, all measurements were conducted in duplicate for quality control purpose. In every session a field blank was obtained at the reference site. During each 2-week session, 12 sites were measured simultaneously including the reference. In total, 16 sessions were performed spread over a 2-year period.

Sampling site criteria were based upon the ESCAPE and VESPA study (www.escapeproject.eu/vespa). Criteria for the site included that the monitoring site had to be on the street side for traffic locations and not placed near exhaust flues or vents.

- 1. *Sampling methods and analyses*

PM_2.5_ was sampled with cyclones (GK 2.05 KTL, BGI inc., Waltham, MA) connected to BGI 400s personal sampling pump units (BGI inc., Waltham, Massachusetts). PM2.5 was sampled on Teflon filters (Zefon international, Ocala Florida). In order to prevent possible overloading of the filter, timers were used to turn the pump on for 5 minutes during every 15 minutes. Thus in two weeks, effectively an-84 h representative sample was taken. The flow of the pump units was measured before and after sampling with calibrated rotameters (Brooks Instruments, Hatfield, Pennsylvania). Elapsed time counters (ETC) of the units were used to record the total sampling time. All samples were prepared and analyzed centrally in Utrecht (IRAS, Utrecht) and cooled during transport. PM_2.5_ concentrations were determined by double pre- and post-weighing of the Teflon filters with a XP2U ultra micro balance (Mettler-Toledo, Greifensee, Switzerland). Filters were stored at 4°C and were weighted after equilibrating for 24 hours in a temperature (20 to 23°C) and humidity (30 to 40%) controlled room, following US EPA criteria and ESCAPE procedures.

The reflectance of these Teflon filters was measured with the Smoke Stain Reflectometer model M43D (Diffusion systems, London UK). The reflectance was transformed into absorbance (a) according to ISO 9835:

a= (A/2V) x ln (R0/Rs)

where A is the loaded filter area (m^2^), V the sampled volume (m^3^), R0 the average reflectance of field blank filters, and Rs the reflectance of the sampled filter.

Absorbance was expressed in 10^-5^ m^-1^. Absorbance correlates well with actual measurements of elemental carbon (soot). We will use the term soot hereafter.

PM_2.5_ measurements were discarded if the elapsed time counter (ETC) showed that the unit had run less than 2/3 of the target of 5040 minutes and/or if the end flow of the pump unit was below 2 L/m. A few high post flows larger than 4.5 l/min were assigned a flow of 4.5 L/minute as it is physically impossible for the pump to support flows substantially larger than 3.5 L/min.

Ogawa diffusion badges (Ogawa & Company USA Inc., Pompano Beach Florida) were used for NO_x_ and NO_2_ sampling. NO_2_ and NO_x_ badges were analyzed using a spectrophotometer based upon the Saltzman method, following ESCAPE procedures. From each batch of 40 badges, four were kept at the IRAS laboratory as lab blanks. These lab blanks were analyzed on the same day as the sampled badges from that batch and their average concentrations were subtracted from the sampled concentrations.

For quality assurance and control the aim was to have 16 duplicates and field blanks. Mean field blank weight changes were subtracted from all sample weights. Mean blank filter reflectance was accounted for in the calculation of the absorbance.

To be able to correct for differences in temporal variation related to weather conditions during sampling periods, a reference site was selected. Measurements were conducted at the reference sites during every 14-day measuring period. The reference site was placed at an urban background site. The site was on the top floor of a high-rise university building (45 m).

## Predictor data

Because we anticipated that it might be difficult to obtain specific GIS data on potential predictors for air pollution spatial variability in this relatively small study area, our primary data collection tool was traffic counting and site characterization for all monitoring sites by trained study personnel. We used the methods applied previously in the ESCAPE study (Eeftens, 2012; Beelen, 2013). Traffic counting was performed for 15 minutes separately for motorcycles, mopeds, cars, heavy duty vehicles and buses separately and next transformed intro daily counts using a formula developed for the Netherlands: traffic count per 15 minutes * 4 * 12 * 1.27 (calculate hourly counts, then daytime counts and then using the factor 1.27 to calculate 24-hour counts, taking into account lower counts during the nighttime hours). The factor 1.27 may not apply in Jakarta, but as for all sites the same formula is applied, this would affect primarily the absolute values and not the ranking sites.

Three GPS measurements were taken at every site and the average was used to characterize the geographical position of the site. We used Google Earth to calculate distances from the front door to the nearest road and the nearest major road and the width of the streets. Population and household density data were available at a neighborhood scale from the Indonesian Bureau of Statistical Center (http://jakarta.bps.go.id). We further evaluated GIS variables based upon global databases used in global NO_2_ and PM_2.5_ models. To incorporate potential indoor sources, we characterized each neighborhood with the average indoor concentration measured in the homes of 50 women, where 3 assessments were further excluded to subject loss to follow up.

## LUR model development

Linear regression models were developed using a supervised stepwise selection procedure, first evaluating univariate regressions of the corrected annual average concentrations with all available potential predictors following procedures used before.^35^ The predictor giving the highest adjusted explained variance (adjusted R^2^) was selected for inclusion in the model if the direction of effect was as defined a priori. We then evaluated which of the remaining predictor variables further improved the model adjusted R^2^, selected the one giving the highest gain in adjusted R^2^, and the a priori chosen direction of effect. Subsequent variables were not selected if they changed the direction of effect of one of the previously included variables. This process continued until there were no more variables with the right direction of effect, which still increased the adjusted R^2^ of the previous model.

As final steps, variables with a p-value above 0.10 were removed from the LUR model. If the Variance Inflation Factor (VIF) was higher than 3 –indicating collinearity-, the variable with the highest VIF was removed and the model re-evaluated. Cook’s D statistics were used to detect influential observations. Cook’s D values above 1 were further examined by assessing the changes in model coefficients on excluding the responsible site. Overall model performance was evaluated by five-fold holdout validation: 20% randomly selected sites were left out from the model and the model re-developed using the remaining 80% of the sites. The Moran’s I statistic was not calculated to indicate spatial autocorrelation of the model residuals.
