# Supplementary material for: The effect of exposure to traffic related air pollutants in pregnancy on birth anthropometry: a cohort study in a heavily polluted low-middle income country
Source: Environ Health. 2023 Feb 27;22:22. doi: 10.1186/s12940-023-00973-0 (PMC9969650; doi:10.1186/s12940-023-00973-0)
Supplement: Supplementary file 2 — Additional file 2: Supplemental Table 1. LUR models for air pollutant concentration assessment. [file 12940_2023_973_MOESM2_ESM.docx]

**Supplemental Table 1.** **LUR models for air pollutant concentration assessment**

| **Outdoor air pollutants** | **LUR models** |
| --- | --- |
| PM_2.5_ (μg/m^3^) | = 74.40 + (1.381 x 10^-4^ x t_motor) + (-2.63 x 10^-3^ x pop) + (4.769 x 10^-3^ x  t_heavyvehicle) |
| Soot (10^-5^ per m) | = 5.466 + (1.489 x 10^-5^ x t_motor) + (1.794 x 10^-3^ x total 500) + (- 2.591 x 10^-2^  x ISA) |
| NO_x_ (μg/m^3^) | = - 8,575 + (3.274 x 10^-4^ x t_motor) + (-7.842 x 10^-3^ x NDVI_1) + (80.78 x  y_c) + (3.441 x 10^-3^ x t_heavyvehicle) + (- 1.799 x 10^-4^ x pop) |
| NO_2_ (μg/m^3^) | = - 6,387 + (2.63 x 10^-4^ x t_motor) + (-6.132 x 10^-3^ x NDVI_1) + (60.22 x  y_c)+ (2.876 x 10^-3^ x min_500) + (- 1.478 x 10^-4^ x pop) |

t_motor: motor cycle density at the nearest street; pop: population density; t_heavyvehicle: heavy vehicle density such as trucks etc. at the nearest street; t_ all refer to systematic field observations (15 minute of traffic counting by a trained technician using a previously developed protocol). All other variables obtained from GIS databases. Total 500: major road length in a 500 m buffer (total refers to all open street map categories considered as major road, ISA: Impervious Surface Area; NDVI: normalized difference vegetation index , y_c: y coordinate of subjects’ house, min_500: road length of minor roads in a 500m buffer.
