# Supplementary material for: The effect of exposure to traffic related air pollutants in pregnancy on birth anthropometry: a cohort study in a heavily polluted low-middle income country
Source: Environ Health. 2023 Feb 27;22:22. doi: 10.1186/s12940-023-00973-0 (PMC9969650; doi:10.1186/s12940-023-00973-0)
Supplement: Supplementary file 3 — Additional file 3: Supplemental Table 2. Correlation between air pollutants concentration at the measurement sites. [file 12940_2023_973_MOESM3_ESM.docx]

**Supplemental Table 2. Correlation between air pollutants concentration at the measurement sites (n = 88)**

|  | PM_2.5_ | Soot | NOx | NO_2_ |
| --- | --- | --- | --- | --- |
| PM_2.5_ | 1 | 0.51 | 0.03 | 0.14 |
| Soot | 0.51 | 1 | 0.16 | 0.14 |
| NO_x_ | 0.03 | 0.16 | 1 | 0.87 |
| NO_2_ | 0.03 | 0.14 | 0.87 | 1 |

all p values < 0.05
