# Supplementary material for: The effect of exposure to traffic related air pollutants in pregnancy on birth anthropometry: a cohort study in a heavily polluted low-middle income country
Source: Environ Health. 2023 Feb 27;22:22. doi: 10.1186/s12940-023-00973-0 (PMC9969650; doi:10.1186/s12940-023-00973-0)
Supplement: Supplementary file 4 — Additional file 4: Supplemental Table 3. Correlation between air pollutants concentration in the cohort. [file 12940_2023_973_MOESM4_ESM.docx]

**Supplemental Table 3. Correlation between modelled air pollutants concentration exposure in**

**the cohort (n= 340)**

|  | PM_2.5_ | Soot | NO_x_ | NO_2_ |
| --- | --- | --- | --- | --- |
| PM_2.5_ | 1 | 0.47** | 0.08** | 0.14* |
| Soot | 0.47** | 1 | 0.23** | 0.30** |
| NO_x_ | 0.08** | 0.23** | 1 | 0.99** |
| NO_2_ | 0.14* | 0.30** | 0.99** | 1 |

*= p< 0.05

** = p< 0.001
