# Supplementary material for: The effect of exposure to traffic related air pollutants in pregnancy on birth anthropometry: a cohort study in a heavily polluted low-middle income country
Source: Environ Health. 2023 Feb 27;22:22. doi: 10.1186/s12940-023-00973-0 (PMC9969650; doi:10.1186/s12940-023-00973-0)
Supplement: Supplementary file 5 — Additional file 5: Supplemental Table 4. The association between exposure to indoor air pollutants during pregnancy with birth anthropometrics in a-indoor subset group. [file 12940_2023_973_MOESM5_ESM.docx]

**Supplemental table 4. The association between exposure to indoor air pollutants during pregnancy with birth anthropometrics in a-indoor subset group (n=47)**

|  | | Linear regression coefficients (95% confidence interval) | |
| --- | --- | --- | --- |
|  |  | **Birth weight (g)** | **Birth length (mm)** |
| PM_2.5_ | Crude | -24.0 (-62.8;14.7) | -0.71 (-2.49;1.07) |
|  | Adjusted | -24.3 (-68.8;20.2) | -0.92 (-3.29;1.44) |
| Soot | Crude | -18.1 (-49.5;13.3) | -0.51 (-1.95;0.93) |
|  | Adjusted | -13.7 (-46.3;19.0) | -0.76 (-2.64;1.12) |
| NO_x_ | Crude | 2.9 (-15.3;21.2) | 0.17 (-0.66;0.99) |
|  | Adjusted | 11.7 (-10.3;33.9) | 0.38 (-0.93;1.70) |
| NO_2_ | Crude | 8.6 (-34.8;32.2) | -0.20 (-1,24;0.84) |
|  | Adjusted | 15.9 (-7.2;38.9) | -0.07 (-1.46;1.32) |

All effect estimates correspond to interquartile increase of each air pollutant i.e. every 7.14 μg/m^3^ for PM_2.5_, 0.75 x 10^-5^

per m for soot, 4.68 μg/m^3^ for NO_x_, and 3.74 μg/m^3^ for NO_2._

Adjusted for gestational age, parity, SES, mother’s working status, mother’s age at pregnancy, delta BMI during pregnancy, infant’s sex, passive smoking exposure, pregnancy complication.
